# Galectin-1 Prevents Infection and Damage Induced by *Trypanosoma cruzi* on Cardiac Cells

**DOI:** 10.1371/journal.pntd.0004148

**Published:** 2015-10-09

**Authors:** Alejandro F. Benatar, Gabriela A. García, Jacqeline Bua, Juan P. Cerliani, Miriam Postan, Laura M. Tasso, Jorge Scaglione, Juan C. Stupirski, Marta A. Toscano, Gabriel A. Rabinovich, Karina A. Gómez

**Affiliations:** 1 Laboratorio de Biología Molecular de la Enfermedad de Chagas (LabMECh), Instituto de Investigaciones en Ingeniería Genética y Biología Molecular (INGEBI-CONICET), Buenos Aires, Argentina; 2 Instituto Nacional de Parasitología “Dr. Mario Fatala Chaben”, Administración Nacional de Laboratorios e Institutos de Salud “Dr. Carlos G. Malbrán”, Buenos Aires, Argentina; 3 Laboratorio de Inmunopatología, Instituto de Biología y Medicina Experimental (IBYME-CONICET), Buenos Aires, Argentina; 4 Hospital Pedro de Elizalde, Servicio de Cardiología, Sección Electrofisiología, Buenos Aires, Argentina; 5 Facultad de Ciencias Exactas y Naturales, Universidad de Buenos Aires, Buenos Aires, Argentina; New York University School of Medicine, UNITED STATES

## Abstract

**Background:**

Chronic Chagas cardiomyopathy caused by *Trypanosoma cruzi* is the result of a pathologic process starting during the acute phase of parasite infection. Among different factors, the specific recognition of glycan structures by glycan-binding proteins from the parasite or from the mammalian host cells may play a critical role in the evolution of the infection.

**Methodology and Principal Findings:**

Here we investigated the contribution of galectin–1 (Gal–1), an endogenous glycan-binding protein abundantly expressed in human and mouse heart, to the pathophysiology of *T*. *cruzi* infection, particularly in the context of cardiac pathology. We found that exposure of HL–1 cardiac cells to Gal–1 reduced the percentage of infection by two different *T*. *cruzi* strains, Tulahuén (TcVI) and Brazil (TcI). In addition, Gal–1 prevented exposure of phosphatidylserine and early events in the apoptotic program by parasite infection on HL–1 cells. These effects were not mediated by direct interaction with the parasite surface, suggesting that Gal–1 may act through binding to host cells. Moreover, we also observed that *T*. *cruzi* infection altered the glycophenotype of cardiac cells, reducing binding of exogenous Gal–1 to the cell surface. Consistent with these data, Gal–1 deficient (*Lgals1* ^*-/-*^) mice showed increased parasitemia, reduced signs of inflammation in heart and skeletal muscle tissues, and lower survival rates as compared to wild-type (WT) mice in response to intraperitoneal infection with *T*. *cruzi* Tulahuén strain.

**Conclusion/Significance:**

Our results indicate that Gal–1 modulates *T*. *cruzi* infection of cardiac cells, highlighting the relevance of galectins and their ligands as regulators of host-parasite interactions.

## Author Summary

Galectins are a family of endogenous lectins defined by a well-conserved carbohydrate recognition domain (CRD) that recognizes β-galactoside-related glycans presented by several glycoconjugates. Up to now, fifteen galectins have been identified in a variety of cells and tissues and proposed to be crucial in diverse biological processes. Galectin–1 (Gal–1), a prototype member of the galectin family, plays key roles in pathogen recognition and in the modulation of innate and adaptive host immune responses. Following infection with the intracellular parasite *Trypanosoma cruzi*, the etiological agent of Chagas disease, Gal–1 was found to be up-regulated in cardiac tissue from patients with chronic Chagas cardiomyopathy. In the present study, we identified a protective role of Gal–1 in *T*. *cruzi* infection of cardiac cells, highlighting the ability of this parasite to control the glycophenotype of these cells. Our data also disclose the relevance of parasite strain-dependent differences in Gal-1-mediated control of *T*. *cruzi* infection *in vivo*. The findings presented here will contribute to delineate the role of Gal-1-glycan interactions during *T*. *cruzi* infection, particularly in the context of heart tissue injury, with critical implications in Chagas disease.

## Introduction

Chagas disease, caused by infection with the protozoan parasite *Trypanosoma cruzi*, represents the main cause of infectious heart disease in Latin America. It is estimated that about 8 to 10 million people worldwide are infected with *T*. *cruzi*, mostly in Central and South America where Chagas disease is endemic [1,2]. In the last decade, an increased number of cases has been well documented in North America, Europe and Western Pacific, mostly because of the influx of immigrants from endemic countries [3–5].

In humans, the acute phase usually occurs with mild signs and symptoms that are not unique to this disease. However, being the cardiac muscle one of the most heavily parasitized tissues, myocarditis characterized by pericarditis, ventricular enlargement, conduction abnormalities and congestive heart failure is consistently observed in acutely infected patients, with an estimated mortality rate of 0.25 to 0.5%, often in children [6,7]. Myocarditis is found during symptomatic acute parasite infection, but also by histopathological examination of heart biopsies in patients with no apparent signs of cardiac disease [8]. In fact, following acute infection, patients enter an asymptomatic phase, which lasts throughout life in the majority of infected subjects. The remaining 30–40% of chronically infected individuals develop cardiac or digestive disorders (megaoesophagus and megacolon), or both, during their lifetime [6]. The cardiac form is the most common and severe manifestation of Chagas disease, causing congestive heart failure, arrhythmias and conduction abnormalities, which often results in sudden death [9–11].

The mechanisms linking the acute and chronic myocardial progression have not yet been clarified. Currently, it is well accepted that the etiology of chagasic cardiomyopathy is multifactorial, suggesting multiple complex interactions between the host and the parasite [12,13]. Several studies revealed that the development of cardiac symptoms is associated with *T*. *cruzi* persistence and its genetic variability, and these effects are controlled by the host immune response, which involves activated T and B lymphocytes, myeloid cells, pro-inflammatory cytokines, cross-reactive antibodies and endogenous lectins [14–17].

Galectin–1, a proto-type member of the galectin family, has the ability to recognize N-acetyllactosamine (LacNAc) residues present in *N*- and *O*-glycans [18,19]. This lectin plays different roles governed not only by its relative concentrations but also by its subcellular compartmentalization [19]. While intracellular Gal–1 controls signaling pathways via protein-protein or protein-glycan interactions, extracellular Gal–1 plays key roles in cell aggregation, cell adhesion to the extracellular matrix and regulation of cell survival, inflammation, immunity and angiogenesis [20–25]. Recently, Seropian and colleagues demonstrated that Gal–1 expression is up-regulated in cardiac cells exposed to hypoxic microenvironments or proinflammatory cytokines, as well as in peri-infarcted area of the mouse heart after experimental acute myocardial infarction (AMI) and in human cardiac tissue from patients with end-stage chronic failure [26]. Furthermore, hearts from mice lacking the Gal–1 gene (*Lgals1* ^*-/-*^) which underwent experimental AMI, showed a higher number of inflammatory cells together with a lower number of regulatory T (T_reg_) cells compared with their wild-type (WT) counterpart. Overall, these findings suggest a potential role of Gal–1 in controlling the inflammatory response in cardiac tissue exposed to internal or external insults [26].

With regards to *T*. *cruzi* infection, Gal–1 has been found to be up-regulated in cardiac tissue from patients with severe chronic Chagas cardiomyopathy. Moreover, an increase frequency of anti-Gal–1 autoantibodies was found to be associated with the severity of cardiac damage during the course of the disease [27]. Whereas low concentrations of Gal–1 increased the number of trypomastigotes (Tulahuén strain) in infected macrophages by diminishing IL–12 production, high concentrations of this lectin promoted macrophages apoptosis and inhibited parasite replication [28]. However, the role of Gal–1 during *T*. *cruzi* infection of cardiac cells has not been yet elucidated. Here we undertook this study to investigate the expression and function of Gal–1 in the adult murine cardiac cell line HL–1 infected with two different phylogenetic discrete typing units (DTUs) of *T*. *cruzi*, namely the Brazil and Tulahuén strains, belonging to TcI and TcVI DTUs, respectively. In addition, we analyzed the impact of endogenous Gal–1 during the course of experimental *T*. *cruzi* infection using the above mentioned *T*. *cruzi* strains, focusing on parasitemia, survival rates and heart alterations. Our findings identify a protective role of Gal–1 on *T*. *cruzi* infection of cardiac cells and demonstrate how parasite infection reprograms expression of cell surface glycans, shifting the balance toward a Gal-1-non-permissive glycophenotype.

## Methods

### Ethics statement

Clinical research protocols followed the tenets of the Declaration of Helsinki. The protocols used in this study were approved by the Medical Ethics Committee of Fernandez Hospital (Buenos Aires, Argentina). All patients gave written informed consent before blood collection and after the nature of the study were explained.

Animal studies were conducted in accordance with the Guide for the Care and Use of Laboratory Animals, 8^th^ Edition (2011). The protocols used were approved by Animal Care Committee of the Instituto Nacional de Parasitología “Dr. Mario Fatala Chaben”, Administración Nacional de Laboratorios e Institutos de Salud “Dr. Carlos G. Malbrán” (Buenos Aires, Argentina).

### Study population

Patient selection was conducted at the Cardiovascular Division of Fernandez Hospital. Positive serology for Chagas disease was determined by two or more tests (indirect immunofluorescence, enzyme-linked immunosorbent assay [ELISA], indirect hemagglutination, or complement fixation) and those patients who had at least two of three reactive serological tests were considered *T*. *cruzi* infected. Patients underwent a complete clinical and cardiologic examination that included medical history, physical examination, electrocardiogram (ECG) at rest, laboratory and chest X-ray examinations, and Doppler echocardiography evolution. The exclusion criteria considered the presence of systemic arterial hypertension, diabetes mellitus, thyroid dysfunction, renal insufficiency, chronic obstructive pulmonary disease, hydroelectrolytic disorders, alcoholism, history suggesting coronary artery obstruction and rheumatic disease, and the impossibility of undergoing clinical examination.

The study population consisted of 28 patients who completed the screening protocol and were in the chronic phase of the infection, 19 patients with cardiac symptoms and 9 patients in the asymptomatic phase. Forty-two non-infected individuals, within the same age range (30–70 years old) which showed negative serological tests for Chagas disease, were included as control group.

### Production of recombinant Gal–1

Recombinant Gal–1 (rGal–1) was produced and purified essentially as described previously [23,29]. LPS content of the purified samples (<60 ng/mg) was tested using a gel-clot Limulus test (Associates of Cape Cod, Falmouth, MA).

### Parasites

Trypomastigotes of the Brazil and Tulahuén (stock Tul–2) strains [30,31] were obtained from the extracellular medium of infected monolayers of Vero cells. After separation of Vero cells and cellular debris by centrifugation at 500 x g for 5 min, trypomastigotes were collected by centrifugation at 2,200 x g for 10 min and resuspended in RPMI medium containing 10% FCS. Parasites were counted using a Neubauer chamber and used for *in vitro* infection experiments as described below.

Bloodstream trypomastigotes of both strains were maintained *in vivo* by serial passages of blood-form trypomastigotes in BALB/c mice.

### Cell cultures and transfection

HL–1, an immortalized adult murine cardiac cell line, was plated onto gelatin/fibronectin pre-coated vessels and cultured in Claycomb medium (Sigma-Aldrich, St. Louis, MO, USA) supplemented with 10% FCS, 100 U/ml penicillin, 100 μg/ml streptomycin and 2 mM L-glutamine as previously described [32], under water jacketed incubator at 37°C, 5% CO_2_.

pcDNA3-Gal–1 expression vector was cloned as previously described [33]. Briefly, HL–1 cells were transiently transfected in a 24-well tissue plate with pcDNA3-Gal–1 plasmid or empty vector (0.45 μg DNA/well) using the Lipofectamine 2000 reagent (Invitrogen Co., Carlsbad, USA), according to the manufacturer’s instructions. Cells were selected for resistance to Geneticine (Life Technologies, Foster City, CA). Transfection efficiency was tested by determining Gal–1 concentration in the supernatants of HL–1 cells by ELISA.

### *T*. *cruzi* trypomastigote invasion assay

HL–1 cells seeded on 12 mm cover-slips in 24-well tissue plates (2 x 10^4^ cells/well), were incubated with rGal–1 (10 or 50 μg/ml) for 24 h, in the presence or absence of 100 mM lactose. Then, cardiac cells were infected with trypomastigotes of the Brazil or Tulahuén strains using two different protocols: a) cells were infected with a parasite-to-host cell ratio of 5:1 for 18 h; unattached parasites were then removed by washing with PBS and cells were kept in culture with fresh medium for additional 30 h or, b) cells were infected with a parasite-to-host cell ratio of 10:1 for 4 h; unattached parasites were removed by washing with PBS and cells were kept in culture with fresh medium for 4 days.

In experiments with pcDNA3-Gal-1-transfected HL–1 cells, we directly infected the cells with trypomastigotes according to protocol a). Of note, no differences were found in the percentage of infected cells between wild-type HL–1 cells and those transfected with the plasmid alone (mock) (S1 Fig).

At the indicated time, cells were washed with PBS, fixed in 2% (w/v) paraformaldehyde/PBS overnight, rinsed and kept for 5 min in 0.02 M glycine/ PBS pH 7.4 to quench reactive groups of the fixative. Each manipulation was preceded by washing the cells three times in PBS. After cell permeabilization with PBS-Triton X–100 1% for 5 min and blocking with PBS-BSA 2%, intracellular parasite were identified by indirect immunofluorescence with sera from *T*. *cruzi* infected mice (dilution 1:1,000) as primary antibody (Ab) and Alexa Fluor 488 conjugated goat anti-mouse IgG (Life Technologies, Foster City, CA) as secondary Ab. Dishes were mounted in Vectashield medium (Vector Labs, UK) containing DAPI and visualized using a fluorescence microscope at a magnification of 200X. Images were acquired using an Olympus DP71 digital camera. The number of cells was determined by using Cell Profiler software (version 2). The percentage of infected cells was determined by counting an average of 3,500 cells in each slide on 3–5 distinct coverslips in randomly selected fields; each sample was tested in three to five replicates, in at least two independent experiments. A cell was considered infected when contained at least one intracellular amastigote.

### Infection *in vivo*

Age- and gender-matched mice with genetic deletion of the gene encoding Gal–1 (*Lgals1* ^*-/-*^) and WT mice with equivalent genetic background (C57BL/6) were kindly provided by Dr. Francoise Poirier (Jacques Monod Institute, Paris, France). Eight-week-old mice were inoculated intraperitoneally with 2,500 bloodstream trypomastigotes of the Brazil or Tulahuén strains resuspended in 200 μl RPMI media. Non-infected *Lgals1* ^*-/-*^ and WT mice (n = 5–15) injected only with RPMI media were used as controls. Parasitemia was determined twice a week from the second to the fifth week of infection by counting parasites in a 5 μl drop of tail vein blood. Results were expressed as the number of trypomastigotes per ml of blood. Survival was recorded daily until 95 dpi.

For histopathological studies, infected and control mice were lightly anesthetized with Avertin (tribromoethanol) before sacrifice by cervical dislocation at 120 and 19 dpi with Brazil and Tulahuén strains, respectively. Hearts and skeletal muscle samples were harvested, fixed in 10% neutral buffered formalin, processed routinely and embedded in paraffin. Five micron thickness sections were stained with hematoxylin and eosin (H&E) and examined at an Olympus DP71 light microscope. The number of parasitized cells per section and the extent of inflammation were recorded on a single blind basis as described previously [34,35]. Briefly, different areas of the heart (atria, ventricular walls and septum) and skeletal muscle sections were evaluated qualitatively and scored according to the distribution (focal, confluent or diffuse) and the extent of inflammation as follows: normal (0); focal or a single inflammatory foci (1); multifocal, non-confluent inflammatory infiltrates (2); confluent inflammation with partial section involvement (3); diffuse inflammation extended through the section (4).

### Annexin V staining

HL–1 cells (1 x 10^4^ cells/well) seeded in a 48-well tissue plate were cultured in the presence of rGal–1 (10 or 50 μg/ml) for 18 h and then infected with 5 x 10^4^ trypomastigotes of the Brazil or Tulahuén strains. After 18 h, cells were washed to remove unattached parasites and incubated for 48 h. Samples were finally processed for phophatidylserine exposure by using the FITC-Annexin V Apoptosis Detection Kit (BD Pharmingen, Chicago, IL, USA) according to the manufacturer’s instructions. Cells fixed with 2% (w/v) paraformaldehyde/PBS, were acquired using a FACSAria flow cytometer. Data were analyzed with WinMdi software.

### Gal–1 binding to *T*. *cruzi*

#### Fluorescence staining

Two hundred μl of RPMI media containing 2.5 x 10^5^ trypomastigotes of the Brazil or Tulahuén strains, were added to round 12 mm coverslips previously treated with poly-L-lysine 10 mM for 30 min, washed with distilled water and air-dried. After 30 min, parasites were fixed in 2% (w/v) paraformaldehyde/PBS overnight at 4°C. Residual formaldehyde was quenched by addition of 0.02 M glycine in PBS pH 7.4. Each manipulation was preceded by washing the parasites three times in PBS. After blocking in PBS-BSA 2%, parasites were treated with rGal–1 (25 μg/ml) for 1 h, followed by incubation with anti-mouse Gal–1 Ab labeled with Alexa Fluor 488 (dilution 1:200 in PBS-BSA 1%). As control, parasites were incubated with a rabbit anti-Tc13 polyclonal Ab (dilution 1:500) for 90 min at room temperature and then, revealed with anti-rabbit IgG Cy3 Ab (dilution 1:200 in PBS-BSA 1%) (Sigma-Aldrich, St.Louis, MO, USA). Dishes were mounted in Vectashield medium (Vector Labs, UK) containing DAPI and visualized using an Olympus BX41 fluorescence microscope at a magnification of 200X. Images were acquired using an Olympus DP71 digital camera.

#### Flow cytometry

Approximately 2.5 x 10^5^ trypomastigotes of the Brazil or Tulahuén strains, were harvested from infected Vero cells, washed twice with 0.5 ml of cold PBS-BSA 1%. After washing three times in 10 mM HEPES, 150 mM NaCl pH 7.4 containing 1% BSA (lectin buffer-BSA 1%), trypomastigotes were incubated with FITC-labeled Gal–1 (25 μg/ml in lectin buffer-BSA 1%). As control, parasites were simultaneously incubated with a rabbit anti-Tc13 polyclonal Ab (dilution 1:1,000) and then revealed with anti-rabbit Ab conjugated with APC (dilution 1:200 in lectin buffer-BSA 1%) (BD Pharmingen, Chicago, IL, USA) for 1 h at room temperature. After washing, parasites were fixed with 2% (w/v) paraformaldehyde/PBS and a minimum of 10,000 events were acquired on a FACSAria flow cytometer (Becton Dickinson). Non-specific binding was determined with streptavidin-FITC only. Data analyses were carried out with WinMdi software.

### Glycophenotype and Gal–1 binding assays

*T*. *cruzi*-infected and non-infected HL–1 cells were harvested at 5 dpi and counted in a Neubauer chamber. Approximately 1 x 10^5^ cells were washed twice with 0.5 ml of cold PBS-BSA 1% and resuspended in lectin buffer-BSA 1%, containing the following biotin-conjugated lectins: PNA, SNA, HPA, LEL, PHA-L, MAL II (S2 Fig and S1 Table) at the appropriate concentration for 1 h at room temperature, and then incubated with FITC conjugated streptavidin (BD Pharmingen, Chicago, IL, USA) in lectin buffer-BSA 1%. After 30 min at room temperature, cells were fixed with 2% (w/v) paraformaldehyde/PBS and a minimum of 10,000 events were acquired on a FACSAria flow cytometer. Non-specific binding was determined with FITC conjugated streptavidin alone. Data analyses were carried out with WinMdi software. Results were expressed as the percentage of positive cells or as the relative specific fluorescence index (SFI) for each lectin. The relative SFI was expressed by the ratio of the SFI of infected cells samples to that of non-infected cells samples; SFI was calculated by dividing mean fluorescence recorded with the specific biotinylated lectin and FITC-streptavidin by the fluorescence intensity obtained with FITC-streptavidin only. Similar procedure was performed to determine Gal–1 binding to HL–1 infected cells and non-infected cells. In this case, cells were incubated with rGal–1 at three different concentrations (5, 10 and 50 μg/ml) in the absence or presence of lactose (100 mM) and further revealed using a FITC-labeled anti-mouse Gal–1 Ab (dilution 1:200 in lectin buffer-BSA 1%).

To confirm the glycophenotype, paraformaldehyde-fixed infected and non-infected cells seeded in 12 mm cover slides were incubated with LEL and PHA-L and FITC conjugated streptavidin as described above. Coverslips were visualized using a fluorescence microscope at a magnification of 200X. Images were acquired using an Olympus DP71 digital camera.

### ELISA

Soluble Gal–1 was determined using an in-house ELISA. Briefly, high binding 96-well microplates (NuncMaxisorb) were coated with capture Ab (2 μg/ml purified rabbit anti-Gal–1 polyclonal IgG) in 0.1 M sodium carbonate pH 9.5. After incubation for 18 h at 4°C, wells were rinsed three times with wash buffer (0.05% Tween–20 in PBS) and incubated for 1 h with blocking solution (2% BSA in PBS). Samples and standards (100 μl) were diluted in PBS-BSA 1% and incubated for 18 h at 4°C. Plates were then washed and incubated with 100 ng/ml biotinylated detection Ab (purified rabbit anti-Gal–1 polyclonal IgG) for 1 h. Plates were rinsed three times before adding horseradish peroxidase-labeled streptavidin (0.33 μg/ml; Sigma-Aldrich, St. Louis, MO, USA) for 30 min at room temperature. After washing, 100 μl of TMB solution (0.1 mg/ml tetramethylbenzidine and 0.06% H_2_O_2_ in citrate-phosphate buffer pH 5.0) was added to the plates. The reaction was stopped by adding 2N HCl and Optical density (OD) was determined at 450 nm in a Versamax microplate reader (Molecular Devices). All samples were tested in duplicate, in two independent experiments. A standard curve ranging from 2.5 to 320 ng/ml of rGal–1 was run in parallel. Results are expressed as means of duplicates, in ng/ml.

### Immunoblot analysis

Infected and non-infected HL–1 cells were washed with PBS, harvested and homogenized in ice-cold lysis buffer (10 mM HEPES, 2 mM EDTA, 150 mM NaCl 150, 0.1% NP40) in the presence of a protease inhibitor kit (Complete Mini EDTA-free, Roche, Germany). After protein quantification by Bradford reagent (Sigma-Aldrich, St. Louis, MO, USA), equal amount of protein (60 μg per lane) was resolved on a 15% SDS-PAGE, transferred to nitrocellulose membranes and then immunoblotted with a rabbit anti-Gal–1 polyclonal Ab (dilution 1:3,000) or a mouse monoclonal Ab for β-actin (BD Pharmingen, Chicago, IL, USA) as a loading control. Blots were then incubated with horseradish peroxidase-conjugated anti-rabbit IgG (Vector Labs, UK) or horseradish peroxidase-conjugated anti-mouse IgG (Sigma-Aldrich, St. Louis, MO, USA). Immunoblots were visualized with the Immobilon chemiluminescent horseradish peroxidase substrate (Millipore, Billerica, MA) according to manufacturer’s instructions. The bands were scanned and quantified using ImageJ software (version 1.410).

### RT-qPCR

Total RNA was extracted using Trizol reagent (Gibco/BRL, Grand Island, USA). Reverse transcription was performed using oligodT and Superscript Reverse II transcriptase (Life Technologies, Foster City, CA), according to the manufacturer’s instructions. Real time RT-PCR was done in Rotor-Gene 6000 (Corbett, UK) device using SYBR Green PCR master mix (Life Technologies, Foster City, CA). Data were analyzed using the relative standard curve method and results were normalized with respect to glyceraldehyde 3-phosphate dehydrogenase (GAPDH) mRNA levels.

The following primers were used: mouse Gal–1, forward 5′-TGAACCTGGGAAAAGACAGC–3′ and reverse 5′-TCAGCCTGGTCAAAGGTGAT–3′; mouse GAPDH forward 5′- ACTCCCACTCTTCCACCT -3′ and reverse 5′- TCCACCACCCTGTTGCT -3′. Expression was calculated from the standard curves and then expressed in arbitrary units of Gal–1 relative to GAPDH.

### Statistical analyses

Unless otherwise indicated, values are expressed as means ± SEM of at least three independent experiments. Statistical comparisons were performed with one-way ANOVA followed by Tukey test for multiple-group comparisons, except for glycophenotype analysis where Dunnett’s test was used to compare every mean with the control mean. For Gal–1 expression determined by Western-blot, Student’s *t* test was used. Concentration of Gal–1 measured by ELISA was evaluated by using non-parametric Kruskal-Wallis test followed by Dunn’s multiple comparison test, while parasitemia and histological findings were analyzed by using the Mann Whitney U test. Log-rank test was performed for statistical comparison of animal survival curves. All tests were performed using GraphPad Prism (GraphPad Software Inc., CA, USA). *p*<0.05 was considered statistically significant.

## Results

### Elevated Gal–1 levels in sera from asymptomatic and symptomatic patients with chronic Chagas Disease

**Fig 1:**
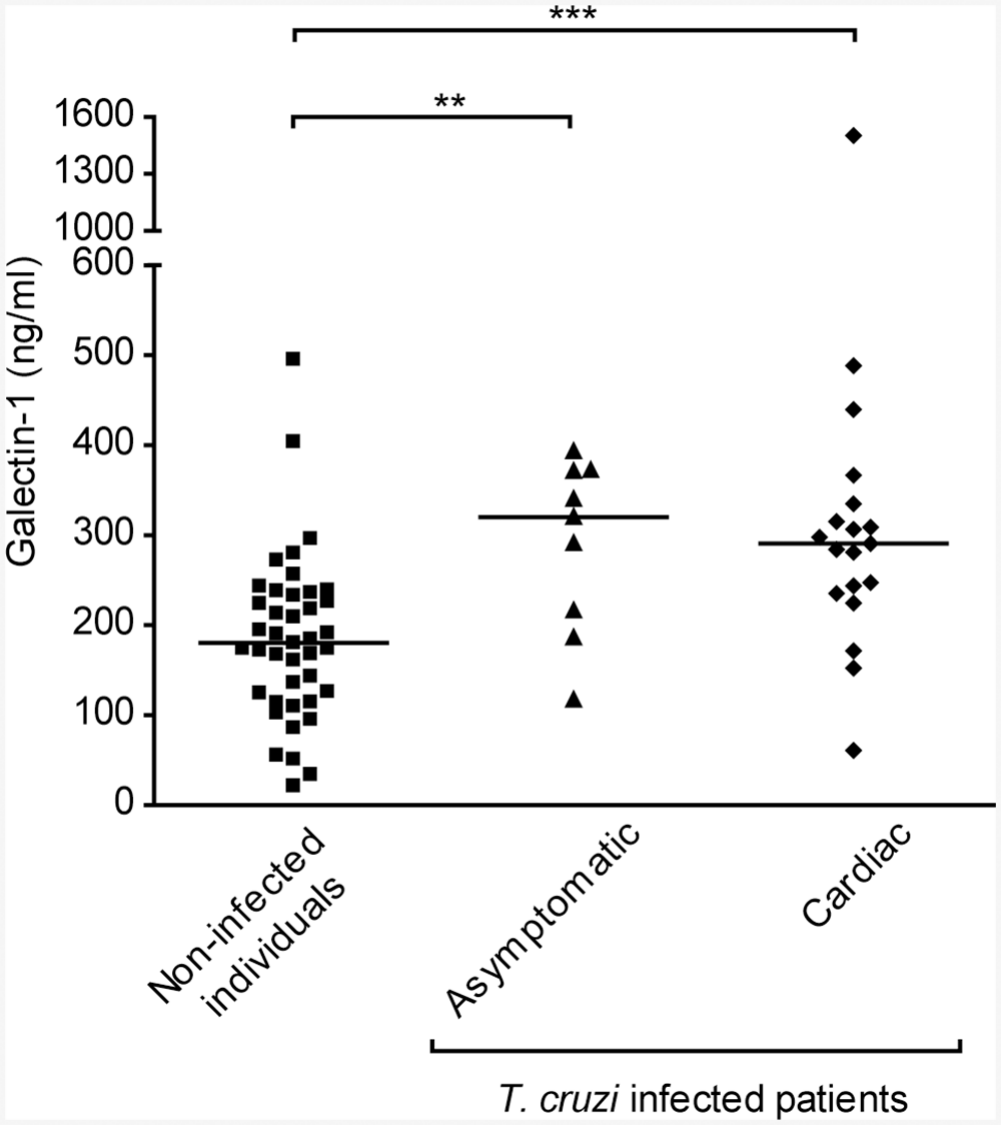
Gal–1 concentration in sera from patients with chronic Chagas disease and non-infected individuals. Serum Gal–1 levels were determined by ELISA, as indicated in the Methods section. Statistical analysis was performed using Kruskal-Wallis test followed by Dunn’s multiple comparison test. **p<0.01; ***p<0.001.

Because Gal–1 expression was higher in heart tissue from patients with chronic Chagas cardiomyopathy who underwent cardiac transplantation [26], we first examined Gal–1 levels in sera from patients during the chronic phase of disease. Results showed that Gal–1 was increased in sera from chronic chagasic patients compared with non-infected subjects (Fig 1). However, no difference was observed between Gal–1 levels in sera from cardiac patients compared with those from asymptomatic individuals. Thus, elevated Gal–1 levels delineate chronic Chagas Disease irrespective of cardiac pathology.

### *T*. *cruzi* infection augments Gal–1 release, but not Gal–1 expression in HL–1 cardiac cells

**Fig 2:**
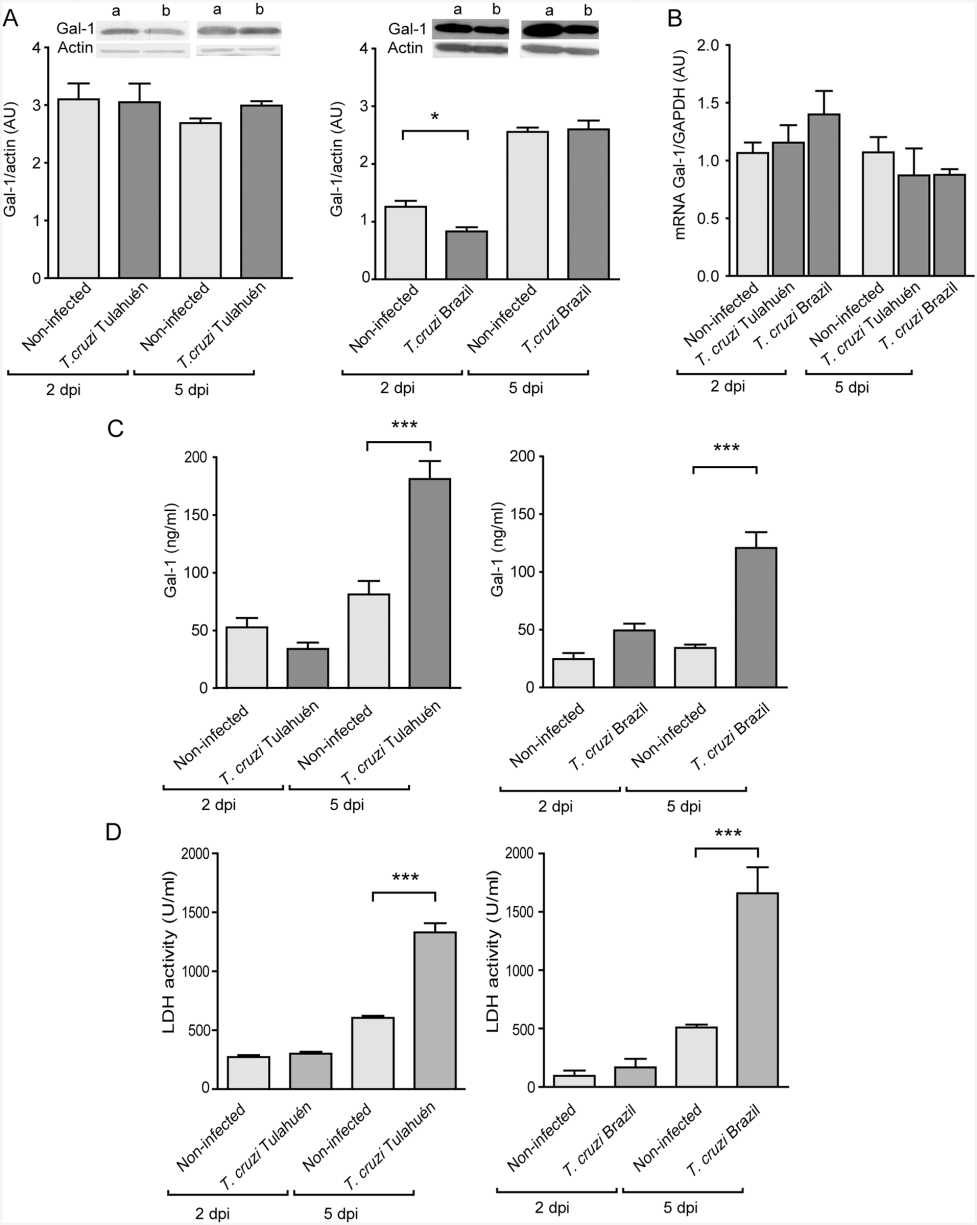
Expression and release of Gal–1 in cultures of HL–1 cells infected with *T*. *cruzi*. Cells were infected with trypomastigotes of Tulahuén or Brazil strains, in a parasite:cell ratio of 5:1, and incubated for additional 2 or 5 days. A) Immunoblot analysis of Gal–1 expression in lysates from non-infected (a) and infected (b) HL–1 cells. Immunoreactive protein bands were semiquantified by densitometry. Results are expressed as Arbitrary Units (AU) relative to β-actin. B) RT-qPCR analysis of Gal–1 mRNA expression of non-infected and infected HL–1 cells. Results are expressed as relative to GAPDH mRNA. C) Detection of Gal–1 in the supernatant of non-infected and infected HL–1 using trypomastigotes of the Tulahuén and Brazil strains, as measured by ELISA. D) Detection of LDH activity in the supernatants of non-infected and infected HL–1 cells by using the LDH-UP kit (Weiner Lab, Argentina), following the manufacturer’s instructions. Results are expressed as Units/ml (U/ml). Data represent the mean ± SEM of three (A and B) and two (C and D) independent experiments. Statistical analysis was performed using Student’s *t* test for data shown in A (a *vs* b) and using one-way ANOVA followed by Tukey test in the remaining experiments. *p<0.05; ***p<0.001.

Given the higher levels of Gal–1 in sera and cardiac tissue in response to *T*. *cruzi* infection, we analyzed the expression of this lectin in infected and non-infected cardiac cells. We infected the murine cardiac cell line HL–1 with trypomastigotes belonging to two different DTUs, Tulahuén (TcVI) or Brazil (TcI) strains. The presence of Gal–1 was assessed after 2 and 5 days post infection (dpi) at the protein and mRNA levels. HL–1 cells infected with *T*. *cruzi* Tulahuén strain did not show any difference in the expression of either Gal–1 mRNA or protein compared with non-infected cells at both times analyzed (Fig 2A and 2B). On the contrary, cells infected with *T*. *cruzi* Brazil strain showed a slight reduction in Gal–1 protein levels only after 2 dpi while no significant differences were achieved at mRNA level at any of the times tested (Fig 2A and 2B). However, when we analyzed the secretion of Gal–1, we found increased amount of this lectin in supernatants of HL–1 cells infected with *T*. cruzi (Tulahuén and Brazil strains) at day 5 post-infection (Fig 2C). To evaluate whether enhanced Gal–1 secretion correlated with cellular lysis, we assessed the release of the cytoplasmatic enzyme lactate dehydrogenase (LDH) into the culture media of infected HL–1 cells. Results showed that LDH activity was considerably greater in supernatants of infected HL–1 cells after 5 dpi using trypomastigotes of both strains (Fig 2D). These data showed an association between Gal–1 and LDH release, suggesting that increased lysis of cardiac cells might contribute to greater Gal–1 secretion.

### Gal–1 mitigates *T*. *cruzi* infection of HL–1 cardiac cells

**Fig 3:**
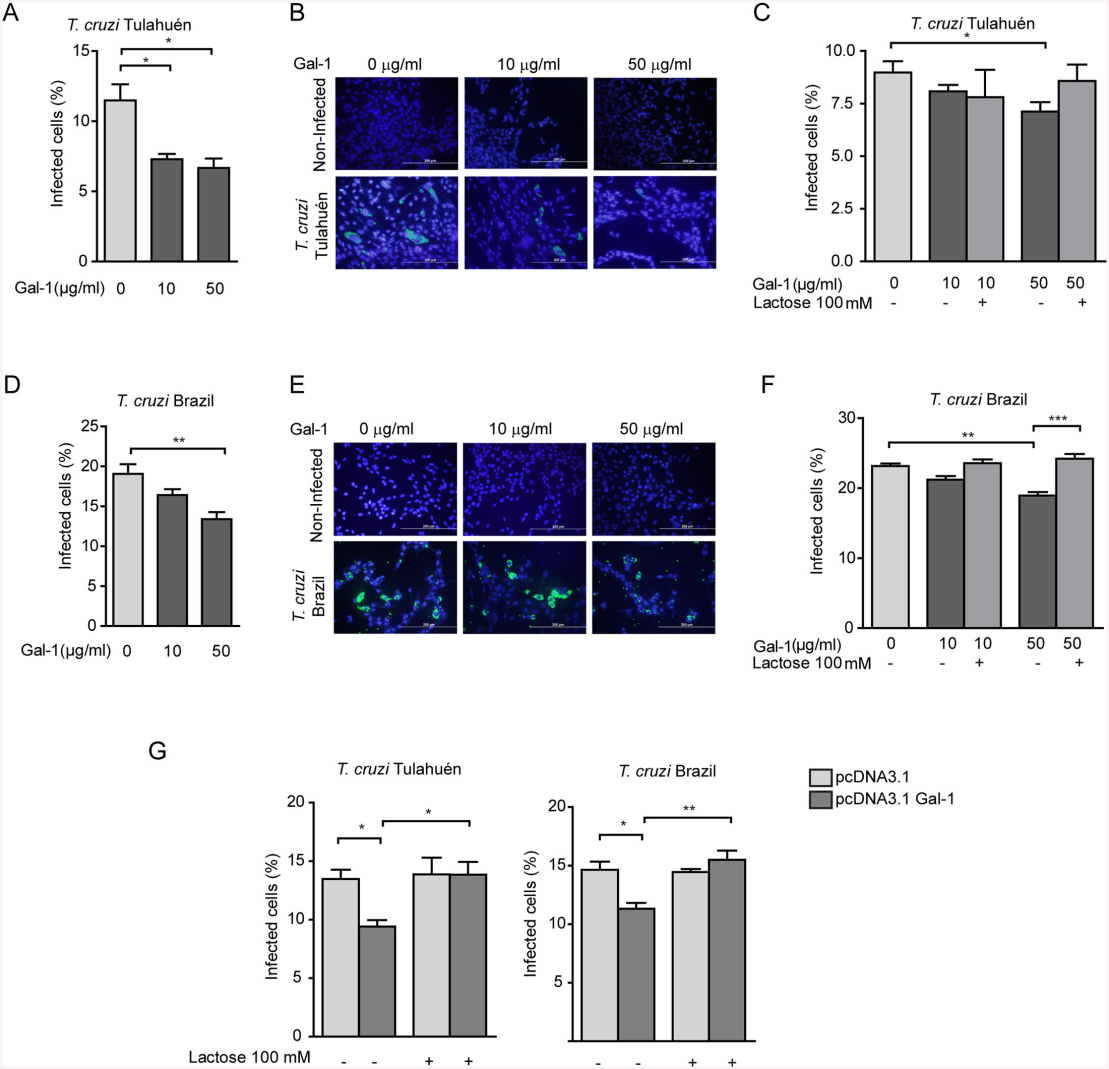
Effect of exogenous rGal–1 in *T*. *cruzi* infection. HL–1 cells were incubated with rGal–1 (10 and 50 μg/ml) for 24 h and then infected with trypomastigotes of both strains. After 4 dpi with *T*. *cruzi* Tulahuén (A) or Brazil strain (D), cells were fixed and stained with an anti-*T*. *cruzi* mouse serum. Representative images are shown in (B) and (E). Similar experiments were performed after 2 dpi with *T*. *cruzi* of the Tulahuén (C) or Brazil strains (F). In this case, some wells were treated with 100 mM lactose, added simultaneously with rGal–1. G) HL–1 cells transfected with pcDNA3-Gal–1 vector or empty vector (mock) were infected with trypomastigotes of both strains, in the presence or absence of 100 mM lactose. Cells were fixed and stained after 2 dpi, with an anti-*T*. *cruzi* mouse serum. In all cases, the percentage of infected cells was determined by counting an average of 3,500 cells in each slide on 3–5 distinct coverslips in randomly selected fields. Results are expressed as mean ± SEM of triplicates determinations from three independent experiments. Statistical analysis was performed using one-way ANOVA followed by Tukey test. *p<0.05; **p<0.01; ***p<0.001.

To investigate the impact of Gal–1 on *T*. *cruzi* infectivity of cardiac cells, HL–1 cells were incubated with different concentrations of recombinant Gal–1 (rGal–1) during 24 h and then infected with trypomastigotes of both strains, Tulahuén and Brazil. Of note, we used rGal–1 doses which did not affect cell viability as evaluated by annexin V-FITC staining (S3 Fig). After 4 dpi with trypomastigotes of the Tulahuén strain, the percentage of infected cells significantly diminished in the presence of increasing concentration of rGal–1 (Fig 3A and 3B). Similar results were obtained when HL–1 cells were infected with the Brazil strain, although a non-significant trend toward a decrease in *T*. *cruz*i infectivity was observed at 10 μg/ml of rGal–1 (Fig 3D and 3E). These data indicate that rGal–1 alters the infection of cardiac cells by *T*. *cruzi*, independently of the parasite lineage (TcI or TcVI). Because at 4 dpi, the percentage of infected cells could be affected by multiple rounds of infection with *T*. *cruzi*, we evaluated the effect of rGal–1 at early time periods of the infection cycle. After 2 dpi, rGal–1 at 50 μg/ml diminished infection of cardiac cells by both *T*. *cruzi* lineages; this effect was prevented in the presence of lactose (Fig 3C and 3F), suggesting that the carbohydrate recognition domain (CRD) of Gal–1 may be involved in Gal–1 modulation of parasite infection. Similar results were observed when Gal–1 transfected HL–1 cells were infected with both *T*. *cruzi* strains (Fig 3G), indicating that both exogenous and endogenous Gal–1 control parasite infectivity.

**Fig 4:**
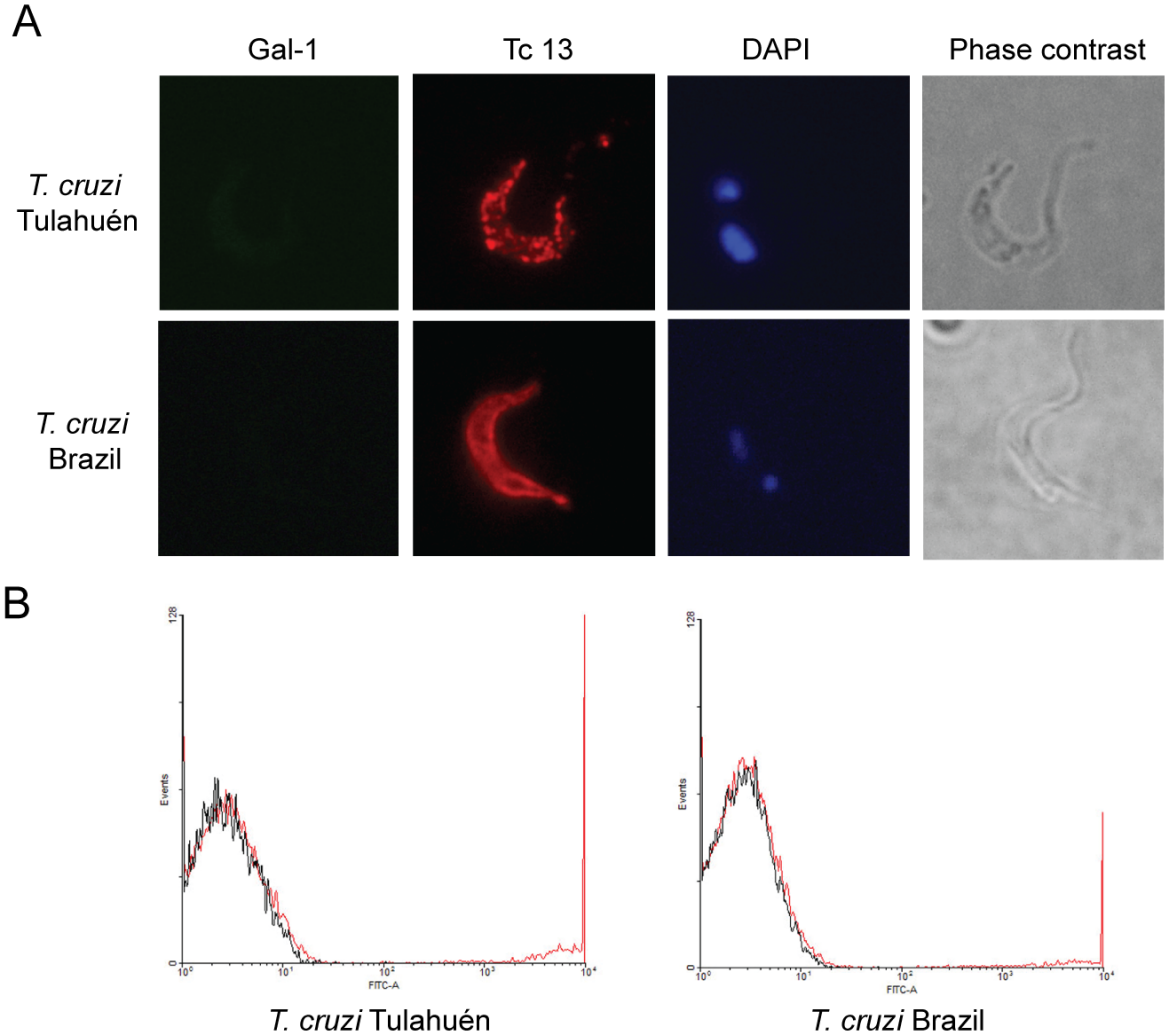
Binding of rGal–1 to *T*. *cruzi* trypomastigotes. A) Fluorescence assay of trypomastigotes incubated with rGal–1 (25 μg/ml) for 1 h, followed by incubation with a mouse anti-Gal–1 Ab labeled with Alexa Fluor 488. Staining with a rabbit polyclonal serum anti-Tc13, a surface protein presented in trypomastigotes, was used as positive control. B) Representative histograms of trypomastigotes of the Tulahuén or Brazil strain incubated with Gal-1-FITC (25 μg/ml). Red lines correspond to parasites treated with Gal-1-FITC, black lines to parasites incubated with streptavidin-FITC used as negative control.

To further analyze the mechanistic bases of this effect, we evaluated whether rGal–1 binds to trypomastigote forms of *T*. *cruzi*, either from Tulahuén or from Brazil strain. Notably, no specific binding of rGal–1 was observed with any of the parasite strains (Fig 4), either by fluorescence staining or by flow cytometry, suggesting that direct binding of Gal–1 to the parasite does not account for the regulatory effects of this lectin.

### Gal–1 prevents *T*. *cruzi* induced phosphatidylserine exposure, an early apoptotic event in HL–1 cells

**Fig 5:**
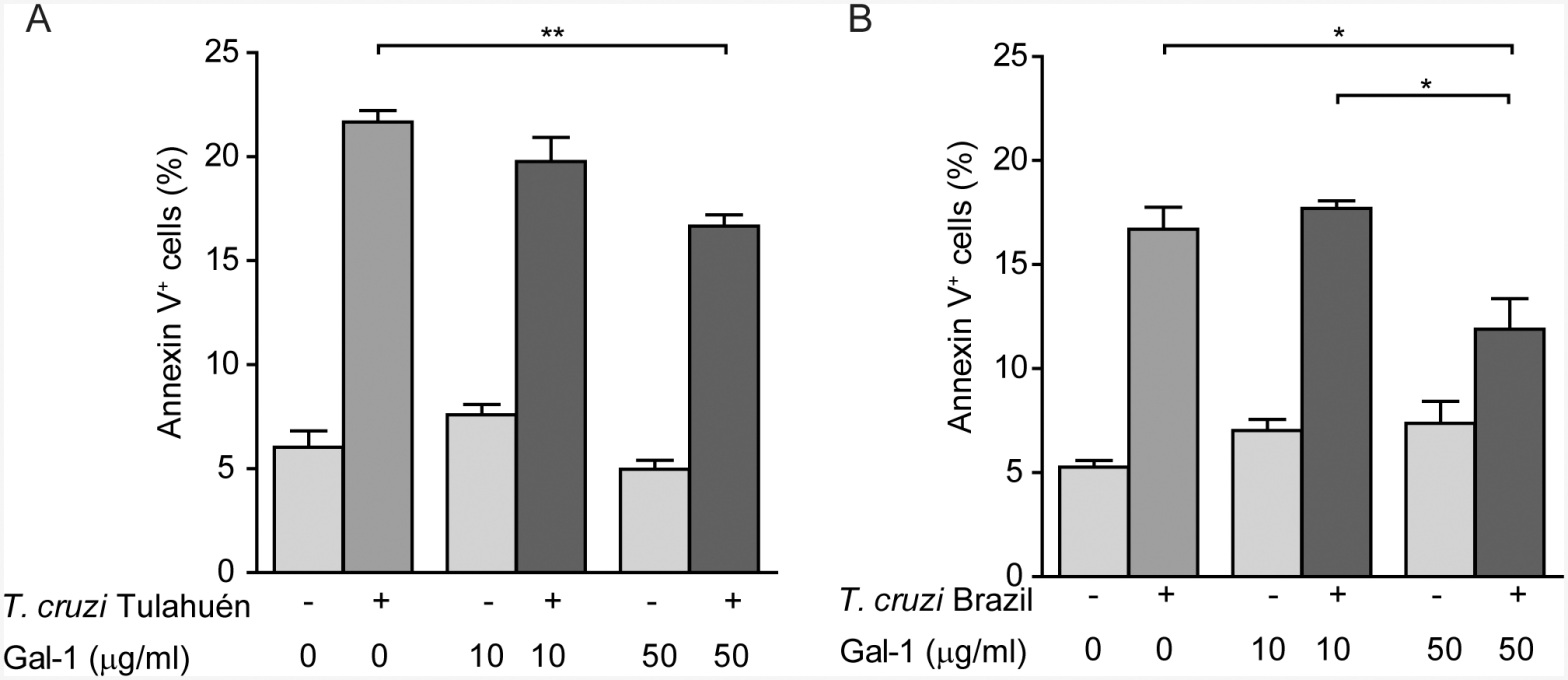
Effect of Gal–1 on phosphatidylserine exposure in *T*. *cruzi* infected HL–1 cells. Cells were incubated with rGal–1 (10 and 50 μg/ml) for 18 h and, then infected with *T*. *cruzi*, Tulahuén (A) or Brazil (B) strains. Annexin V assay was performed at 3 dpi. Results expressed as mean ± SEM are representative of two independent experiments. Statistical analysis was performed using one-way ANOVA followed by Tukey test. *p<0.05; **p<0.01. Only comparisons between infected groups were shown.

The ability of *T*. *cruzi* trypomastigotes to control apoptotic programs in cardiac cells [36,37] and the ability of Gal–1 to regulate viability of different cell types [18], prompted us to investigate the effect of this lectin in parasite induced phosphatidylserine (PS) exposure, an early apoptotic event, on plasma membrane of HL–1 cardiac cells. Thus, we pre-incubated HL–1 cells with rGal–1 (10 and 50 μg/ml) and, following infection with trypomastigotes of the Tulahuén or Brazil strain, annexin V staining was performed. While trypomastigotes of both strains induced considerably exposure of phosphatidylserine residues in HL–1 cardiac cells, the percentage of annexin V-positive cells significantly diminished when cells were pre-incubated with 50 μg/ml rGal–1 before parasite infection (Fig 5). These data suggest that Gal–1, not only reduces *T*. *cruzi* infectivity, but also protects cardiac cells from *T*. *cruzi* driven-phosphatidylserine exposure.

### *T*. *cruzi* induces a Gal–1 resistant glycophenotype in HL–1 cardiac cells

**Fig 6:**
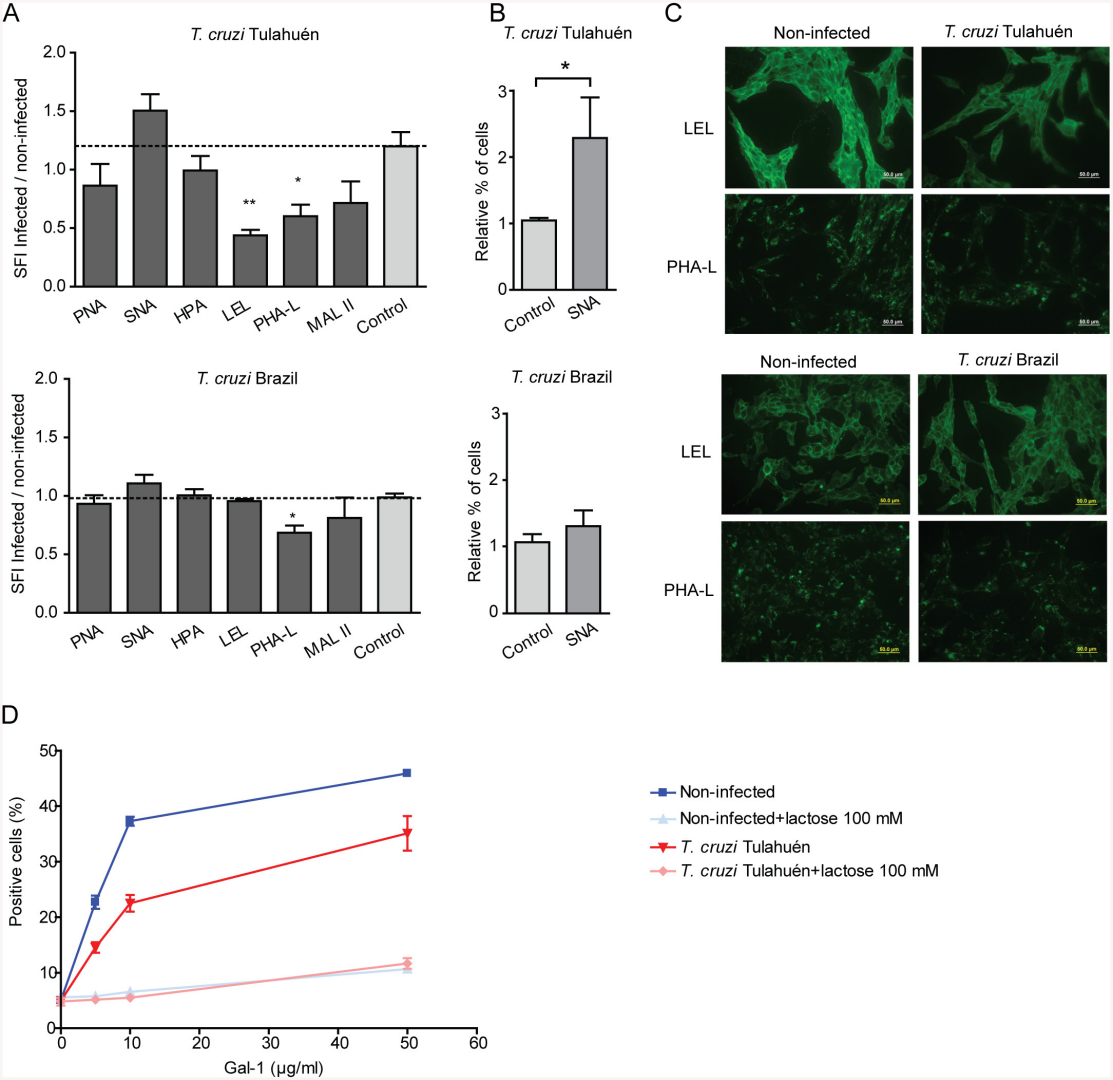
Glycophenotypic analysis of HL–1 cells infected with *T*. *cruzi* trypomastigotes. Non-infected and infected HL–1 cells at 5 dpi with *T*. *cruzi* Tulahuén and Brazil strain, were incubated with different biotinylated lectins (see S1 Table), followed by incubation with FITC-streptavidin. After fixation, cells were analyzed by flow cytometry (A). Results are expressed as relative SFI, calculated as SFI of infected HL–1 cells/SFI of non-infected HL–1 cells for each lectins tested. SFI for each lectin was calculated as the ratio of the mean fluorescence of each samples stained with biotinylated lectin plus streptavidin-FITC over the mean fluorescence of the samples incubated with streptavidin-FITC alone. Dot lines show the mean value of relative SFI of infected over non-infected HL–1 cells incubated with streptavidin-FITC (baseline). Relative percentage of positive cells (infected/non-infected) for SNA staining is shown in B). Results are expressed as mean ± SEM of three independent experiments. Statistical analysis was performed using one-way ANOVA followed by Dunnett´s test. *p<0.05; **p<0.01. Glycophenotypic analysis of infected HL–1 cells was also determined by fluorescence assay. Representative images of LEL and PHA-L staining are shown in C). Binding curve of rGal–1 to HL–1 cells infected with *T*. *cruzi* trypomastigotes of the Tulahuén strain (D). Non-infected and infected cells at 5 dpi with *T*. *cruzi* Tulahuén strain, were incubated with different concentration of rGal–1, in the absence or presence of lactose (100 mM) and then revealed with FITC labeled anti-mouse Gal–1 Ab. After 30 min, cells were fixed and a minimum of 10,000 events were acquired on a FACSAria flow cytometer. Nonspecific binding was determined with FITC conjugated streptavidin alone.

To test if *T*. *cruzi* infection induces changes in the glycophenotype of cardiac cells, we analyzed binding of a panel of biotinylated lectins that recognize specific glycan structures (S2 Fig and S1 Table) to HL–1 cells. Infection with trypomastigotes of the Tulahuén strain led to reduced binding of *Lycopersicon Esculentum* agglutinin (LEL), a lectin that recognizes poly-LacNAc-enriched glycans and phytohemagglutinin-L (PHA-L), a lectin that binds to β1,6-N-acetylglucosamine-branched complex *N*-glycans (Fig 6A). Analysis by fluorescence microscopy confirmed these findings (Fig 6C), indicating that poly-LacNAc and complex branched *N*-glycans, which are key saccharide ligands required for Gal–1 binding, are hindered in response to *T*. *cruzi* infection. Next, we analyzed reactivity for *Sambucus nigra* agglutinin (SNA), a lectin that recognizes α2,6-linked sialic acid. Results showed that the number of SNA^+^ HL–1 cells was significantly higher after infection with trypomastigotes of the Tulahuén strain compared to non-infected cells (Fig 6B).

Gal–1 preferentially recognizes poly-LacNAc units present on the branches of *N*- and *O*-glycans, but does not bind to α2,6-sialylated LacNAc residues [18]. Hence, we hypothesized that increased α2,6-linked sialic acid together with reduced poly-LacNAc and β1,6-branched N-glycans may limit Gal–1 binding to the surface of infected cardiac cells. To address this question, we analyzed binding of rGal–1 to HL–1 cells infected with *T*. *cruzi* Tulahuén strain, in the absence or presence of the galectin-specific disaccharide lactose. Consistent with the surface glycophenotype of these cells, we detected lower binding of Gal–1 to infected versus non-infected cardiac cells; an effect which was dose- and saccharide-dependent (Fig 6D). However, infection of HL–1 cells with trypomastigotes of the Brazil strain led only to reduction of PHA-L reactive complex *N*-glycans, although no changes were detected in the percentage of cells exposing α2-6-linked sialic acid on *N*-glycans (Fig 6A, 6B and 6C). Thus, infection with *T*. *cruzi* Tulahuén strain selectively controls the glycosylation signature of cardiac cells. This strain-dependent regulatory effect may control Gal–1 binding, parasite infection and cardiomyocyte function.

### The protective role of Gal–1 on *T*. *cruzi* infection *in vivo* is dependent on parasite lineage

To investigate the relevance of Gal–1 during *T*. *cruzi* infection *in vivo*, *Lgals1* ^*-/-*^ and WT female and male mice were examined for parasitemia, survival and histopathology, following intraperitoneal inoculation of the parasite.

**Fig 7:**
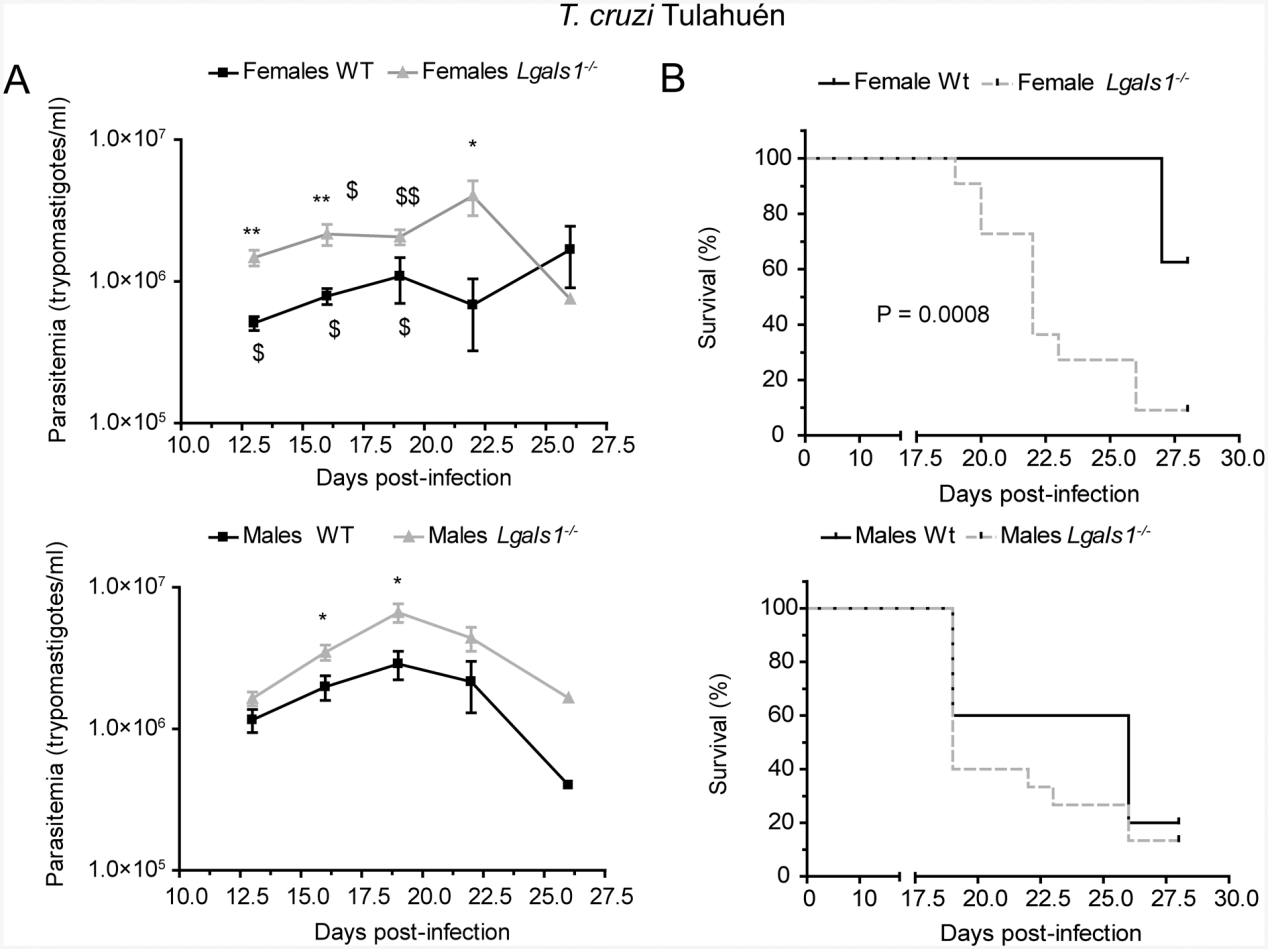
Parasitemia levels (A) and survival rate (B) of WT and *Lgals1* ^*-/-*^ mice acutely infected with *T*. *cruzi* Tulahuén strain, via the intraperitoneal route. For parasitemia levels, each point represents the mean ± SEM of 5–15 animals per group, and statistical analysis was performed using Mann-Whitney U test. *p<0.05, **p<0.01 *vs*. WT mice; ^$^ *p*<0.05, ^$$^ *p*<0.01 *vs*. male mice. For survival rate, statistical analysis was achieved with Log-rank test.

Although mice were challenged with the same parasite inoculum size, the course of the infection was different according to the *T*. *cruzi* strain used. The peak of parasitemia in Tulahuén strain-infected mice (occurring at 19–22 dpi) was higher than that recorded in mice infected with the Brazil strain (at 26–28 dpi; *p*<0.05), suggesting strain-dependent differences in the *in vivo* infectivity of *T*. *cruzi* trypomastigotes (Fig 7A and S4 Fig). Interestingly, *Lgals1* ^*-/-*^ mice infected with trypomastigote of the Tulahuén strain had significantly higher parasitemias compared to WT mice (*p*<0.05), regardless of the gender of the animals (Fig 7A). On the contrary, levels of parasitemia were slightly higher in *Lgals1* ^*-/-*^ male mice infected with the Brazil strain compared to that of WT mice at 28 dpi, whereas no differences were found in female mice (S4 Fig).

The percentage of female *Lgals1* ^*-/-*^ mice that survived acute infection with *T*. *cruzi* Tulahuén strain was significantly lower (P = 0.0008; Fig 7B) and the mean survival time was shorter (P = 0.0127; S2 Table) compared to their WT counterpart. There were no differences in mortality rates and survival time between male *Lgals1* ^*-/-*^ and WT animals infected with trypomastigotes of the Tulahuén strain. On the other hand, mortality rates and survival time of *Lgals1* ^*-/-*^ mice infected with the Brazil strain were similar to that of their WT mice counterparts, regardless of mice gender (S4 Fig and S2 Table).

**Fig 8:**
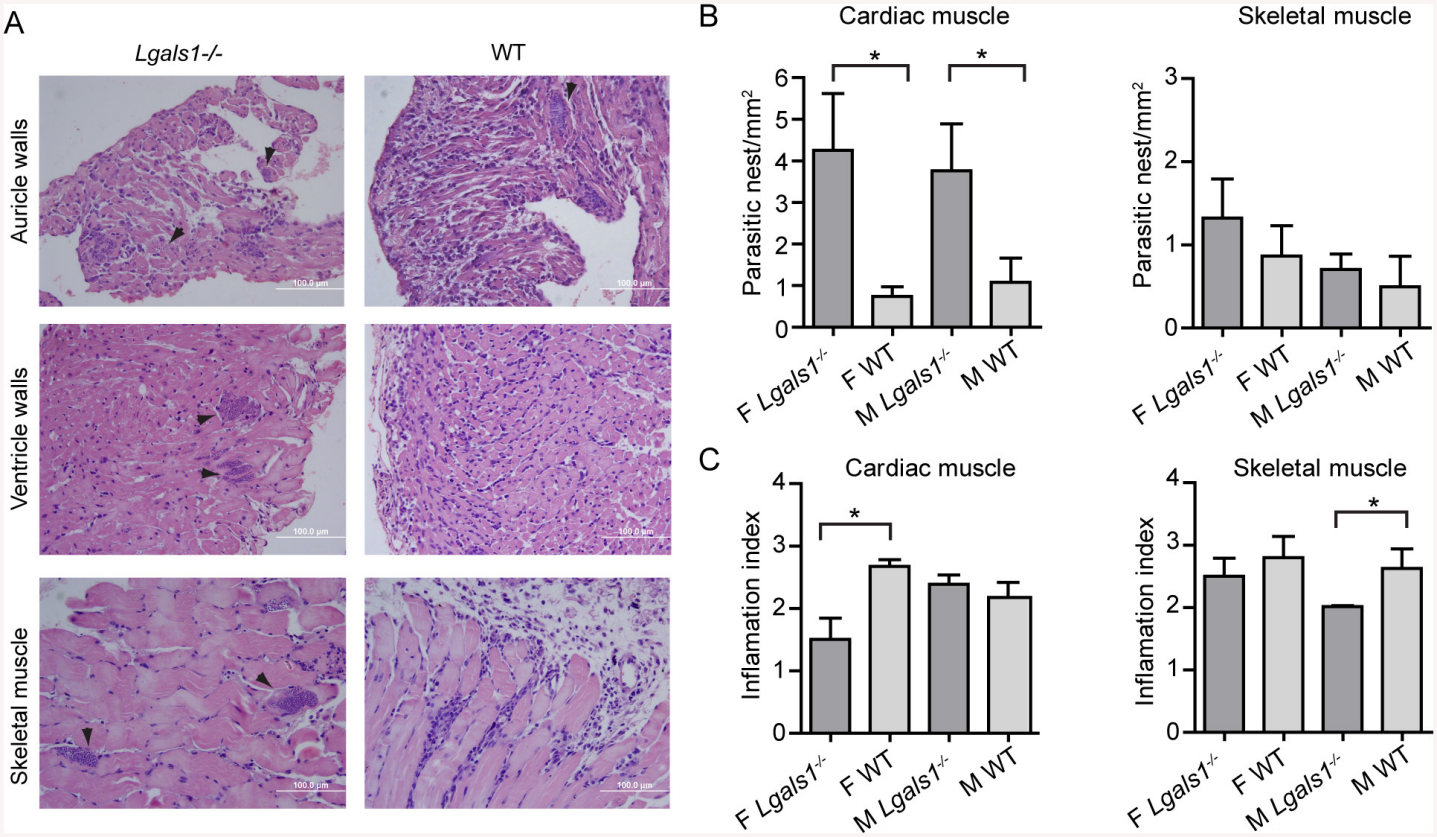
Histopathological findings in *Lgals1* ^*-/-*^ and WT mice at 19 dpi with *T*. *cruzi* Tulahuén strain. A) Microphotographs representative of heart and skeletal muscle histopathological abnormalities (H&E). Parasite density (B) and Inflammation Index (C) were calculated as indicated in the Methods section. Bars represent mean ± SEM of 5–7 mice per group. Statistical analysis was performed using Mann-Whitney U test. *p<0.05. F: Female mice; M: Male mice.

Differences in the survival rates and parasitemia in mice infected with *T*. *cruzi* Tulahuén strain, prompted us to analyze the histopathology of heart and skeletal muscles. The density of parasitized cells was significantly higher in the heart, but not in the skeletal muscle of *Lgals1* ^*-/-*^ mice infected with *T*. *cruzi* Tulahuén strain at the peak of parasitemia, compared with WT mice; this effect was independent of the gender of the mice (*p*<0.05; Fig 8A and 8B). However, the inflammation score in *Lgals1* ^*-/-*^ mice was lower in the heart of females and in the skeletal muscle of male animals, compared with their WT counterparts (Fig 8A and 8C). Because the susceptibility or resistance to infection relies not only on the host but also on parasite genetics, we believe that, in our hands, Brazil strain had less infectivity for C57BL/6 mice and, probably higher inoculum of Brazil strain would recapitulate the results obtained with a more virulent *T*. *cruzi* strain such as Tulahuén. Overall, our data indicate strain-dependent differences in Gal-1-mediated protection of *T*. *cruzi* infection *in vivo*.

## Discussion

The interaction between parasite and host cells is crucial for *T*. *cruzi* survival involving the recognition of a large number of ligands and/or receptors on the surface of both the parasite and the host cells [37–41]. With regards to cardiomyocytes, carbohydrate residues of membrane glycoconjugates, like galactosyl, mannosyl, and sialyl residues, together with mannose receptors not only participate in parasite entry but are also regulated by the infection itself [41]. Intracellularly, the parasite takes control of host cells, including cardiomyocytes which respond to infection with the production of cytokines, chemokines, metalloproteinases, and glycan-binding proteins [41].

Galectins, a family of glycan-binding proteins, have recently emerged as novel regulators of host-parasite interactions [42,43]. With regards to *T*. *cruzi* infection, Pineda *et al*. recently showed the differential recognition by human Gal–1, -3, -4, -7 and -8 of fourteen different strains of *T*. *cruzi* corresponding to the six lineages representing the genetic diversity of the parasite, suggesting strain-dependent glycosylation of the parasite surface [44]. Interestingly, Gal–3, the best studied galectin in the context of *T*. *cruzi* infection, is recruited during parasite invasion of host cells and influences intracellular trafficking of amastigotes [45]. Moreover, expression of Gal–3 in the thymus of *T*. *cruzi* infected mice has been shown to determine the premature exit of immature T cells by modulating thymocyte-extracellular matrix interactions [46]. This ‘chimera-type’ galectin has been found to be required for *T*. *cruzi* adhesion to human coronary artery smooth muscle cells and for B cell function following infection with the parasite [47–49]. In addition, mice lacking Gal–3 showed increased blood parasitemia and impaired cytokine production during *T*. *cruzi* infection [50]. Although the role of other members of the galectin family during *T*. *cruzi* infection still needs to be addressed, these data highlight the multifunctional role of these lectins in host-parasite communication.

Here, we aimed at dissecting the role of Gal–1 and its specific glycans in the infection of cardiac cells with *T*. *cruzi* trypomastigotes (lineages Tcl and TcVI). We demonstrated that Gal–1 not only reduced infection by *T*. *cruzi* but also diminished phosphatidylserine exposure, an early apoptotic event driven by the parasite on HL–1 cells. This effect was also reflected by -*in vivo* experiments showing that *Lgals1* ^*-/-*^ mice intraperitoneally inoculated with *T*. *cruzi* Tulahuén strain had higher parasitemia and lower survival rates than WT animals. Moreover, our data show that *T*. *cruzi* infection can reprogram the glycophenotype of cardiac cells toward a Gal–1 resistant profile, thus highlighting a potential parasite strategy to avoid the beneficial inhibitory effects of Gal–1 in host cells. This Gal–1 restrictive glycophenotype is similar to that observed in T helper (Th)-2 polarized cells [23], in M2-type microglia [51] and in tumor-associated endothelial cells [25].

Regarding the mechanisms underlying the effects of Gal–1 on cardiac cells, it is well known that galectins can act by blocking or stimulating pathogen attachment and infection through binding to host or microbial glycans, or by interfering with molecular interactions required for microbial entry to host cells [42]. In this study, we found, both by flow cytometry and fluorescence staining, that Gal–1 did not bind to *T*. *cruzi* surface glycans from any of the parasite strains analyzed. Therefore, we hypothesized that Gal–1 released via an autocrine or paracrine pathway might recognize specific glycans on the surface of HL–1 cardiac cells that are necessary for *T*. *cruzi* attachment and/or invasion. However, Gal–1 could also act directly facilitating cytokine release by cardiac cells [42].

To determine whether cardiac cells are a major source of Gal–1 production, we evaluated Gal–1 mRNA and protein expression in infected HL–1 cells. Even tough, *T*. *cruzi* infection did not induce Gal–1 expression in HL–1 cells, we observed increased amount of this lectin in culture media of infected cells, probably due to cellular damage generated by parasite release. This result might explain the up-regulation of Gal–1 in heart tissue from patients with chronic Chagas cardiomyopathy as reported by Giordanengo *et al*. [27]. In addition, we found that Gal–1 levels were greater in sera from patients with chronic Chagas disease, irrespective of cardiac alterations. Interestingly, *T*. *cruzi* infection up-regulated Gal–1 expression and secretion in different immune cells, including B cells and macrophages [28, 52].

*In vivo* studies confirmed the protective effect of Gal–1 on *T*. *cruzi* infection observed in the *in vitro* assays. *Lgals1* ^*-/-*^ mice infected with *T*. *cruzi* Tulahuén strain showed higher parasitemia together with lower survival rate, which was more evident in female than male animals. In addition, histopathological analysis revealed a major number of parasites in the heart of those animals, but surprisingly *Lgals1* ^*-/-*^ mice showed a slightly decrease in the inflammatory response as compared to their WT counterparts. Based on previous reports [53], we would expect that a strong inflammatory response will be accompanied by an increased parasite burden in the heart or skeletal muscle of *Lgals1* ^*-/-*^ mice. However, similar findings were reported in mice with genetic or acquired deficiencies of the immune system [35, 54, 55], supporting the notion that the lower inflammation observed in hearts of *Lgals1* ^*-/-*^ mice may be the result of a differential regulation of the immune responses in these knock-out animals.

In addition, differences observed between genders are not unexpected; the immune response to some microorganisms and the subsequent clinical outcome of the infection are linked to host hormonal pathways [56, 57]. Interestingly, substantial disparities in male and female individuals have been clearly documented in *T*. *cruzi* infection [58, 59]. Our results highlight the role of Gal–1 in the complex parasite-driven immune-endocrine networks since important discrepancies in parasitemia and survival rates were observed in male and female animals lacking the *Lgals1* gene. Moreover, in a very recent study, Poncini and colleagues demonstrated that *Lgals1* ^*-/-*^ mice infected by intradermoplantar inoculation with *T*. *cruzi* RA strain, displayed lower mortality and parasite burden in muscle tissue than WT mice [60]. The discrepancy with our data could be associated with different administration routes as the presence of different phagocytic cell types at sites of inoculation and the local immune response triggered by *T*. *cruzi* infection may dictate not only changes in parasite load but also susceptibility or resistance to infection [61–63]. Altogether, these findings suggest that galectin-glycan interactions may influence the outcome of the infection depending on the strain of the parasite (Tulahuén, Brazil or RA), the route of infection (intraperitoneal or intradermoplantar inoculation) and the subtle differences in the immune responses triggered by each *T*. *cruzi* strain [61–63]. Furthermore, similar strain-specific divergences have been previously reported by Toscano *et al*., showing that endogenous Gal–3 can differentially regulate the outcome of experimental malaria, when three distinct strains of rodent malaria parasites, *Plasmodium yoelii 17XNL*, *Plasmodium berghei ANKA* and *Plasmodium chabaudi AS* were inoculated in mice lacking Gal–3 [64].

Finally, our data suggest that the parasite may display evasive mechanisms to counteract the effect of Gal–1. In fact, *T*. *cruzi* infection altered the glycophenotype and decreased the availability of galectin-binding sites on cardiac cells as evidenced by increased α2-6-sialylation which prevented Gal–1 recognition of poly-LacNAc structures. Changes in the glycophenotype were not as evident in HL–1 cells infected with parasites belonging to the Brazil strain, which could explain, at least in part, the differential infectivity of this strain compared with the Tulahuén strain. In line with these findings, Vray *et al*. described that changes in glycosylation structures of infected dendritic cells rendered a profile that was more reactive with Gal–3, affecting not only the infectivity, but also the migratory capacity of these cells in the context of Chagas disease [65]. In this regard, it has been demonstrated that electric communication in the heart is modulated by regulated glycosylation, particularly by sialylation of the cardiac voltage-gated Na^+^ channels (Nav) and Kv [66–68]. Our results suggest that parasite-induced remodeling of the host cell glycome might contribute to mechanical and functional alterations in the heart of infected host.

In conclusion, our findings demonstrate that: a) Gal–1 inhibits *T*. *cruzi* infection of cardiac cells, and b) parasite infection alters the surface glycophenotype of cardiac cells, restricting Gal–1 and possibly limiting its inhibitory activity. Importantly, these effects were dependent on multiple parameters including parasite inoculum and strain, route of entry of the parasite and gender of the host. Thus, modulation of Gal-1-glycan interactions in cardiac cells may influence parasite-induced heart injury. Further studies are warranted to clarify the potential clinical relevance of our findings.

## Supporting Information

S1 Fig Infection of HL–1 cells wild-type and transfected with pcDNA 3.1 plasmid (mock). Cells infected with trypomastigotes of Tulahuén strain, were fixed and stained after 2 dpi with an anti-*T*. *cruzi* mouse serum. The percentage of infected cells was determined by counting an average of 3,500 cells in each slide on 4 distinct coverslips in randomly selected fields. Results are expressed as mean ± SEM. Statistical analysis was performed using Student´s *t* test.
(TIF)

S2 Fig Schematic representation of *N-* and *O*-glycans and lectin-binding sites. MAL II: *Maackia amurensis* agglutinin II; S*NA*: *Sambucus nigra* aglutinin¸ LEL: *Lycopersicon esculentum* agglutinin; *PHA-L*: Phytohemagglutinin-L; *HPA*: *Helix pomatia* agglutinin; Gal–1: galectin–1.
(TIF)

S3 Fig HL–1 apoptosis induced by different rGal–1 concentrations. HL–1 cells were incubated with rGal–1 for 18 h, staining with FITC-Annexin-V and processed by flow cytometry. Results expressed as mean ± SEM, are representative of 2 independent experiments. Statistical analysis was performed by using ANOVA one-way followed by Tukey. **p<0.01.
(TIF)

S4 Fig Parasitemia levels (A) and survival rate (B) of WT and *Lgals1* ^*-/-*^ mice infected with *T*. *cruzi* Brazil strain, via the intraperitoneal route. For parasitemia levels, each point represents the mean ± SEM of 5–15 animals per group, and statistical analysis was performed using Mann-Whitney U test. *p<0.05 *vs*. WT mice; ^$$^ *p*<0.01 *vs*. male mice. For survival rate, statistical analysis was achieved with Log-rank test.
(TIF)

S1 Table Glycan-binding specificity of the lectins used for glycophenotyping. ^1^Concentration of the lectins used.
(DOCX)

S2 Table Groups of mice were injected i.p. with 2,500 bloodstream trypomastigotes of the Tulahuén or Brazil strains. ^a^Parasitemia is shown as median (rank) of parasites/ml of blood from 5 to 15 mice per group of each gender, at the peak. ^b^Survival time is shown as means ± SEM. ND: not detectable.
(DOCX)
