# Supplementary figures and images for: Galectin-1 Prevents Infection and Damage Induced by Trypanosoma cruzi on Cardiac Cells
Source: PLoS Negl Trop Dis. 2015 Oct 9;9(10):e0004148. doi: 10.1371/journal.pntd.0004148 (PMC4599936; doi:10.1371/journal.pntd.0004148)

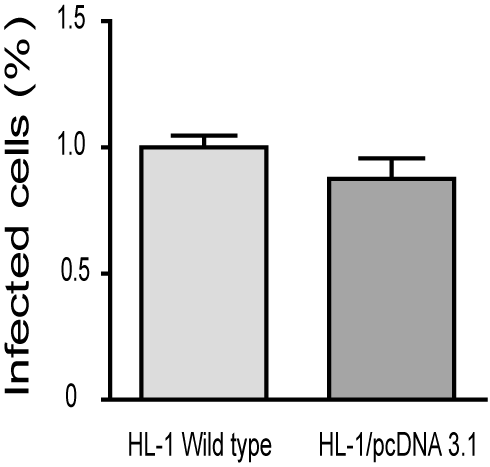

Supplement: S1 Fig — Cells infected with trypomastigotes of Tulahuén strain, were fixed and stained after 2 dpi with an anti-T. cruzi mouse serum. The percentage of infected cells was determined by counting an average of 3,500 cells in each slide on 4 distinct coverslips in randomly selected fields. Results are expressed as mean ± SEM. Statistical analysis was performed using Student´s t test. (TIF) [file pntd.0004148.s001.tif]

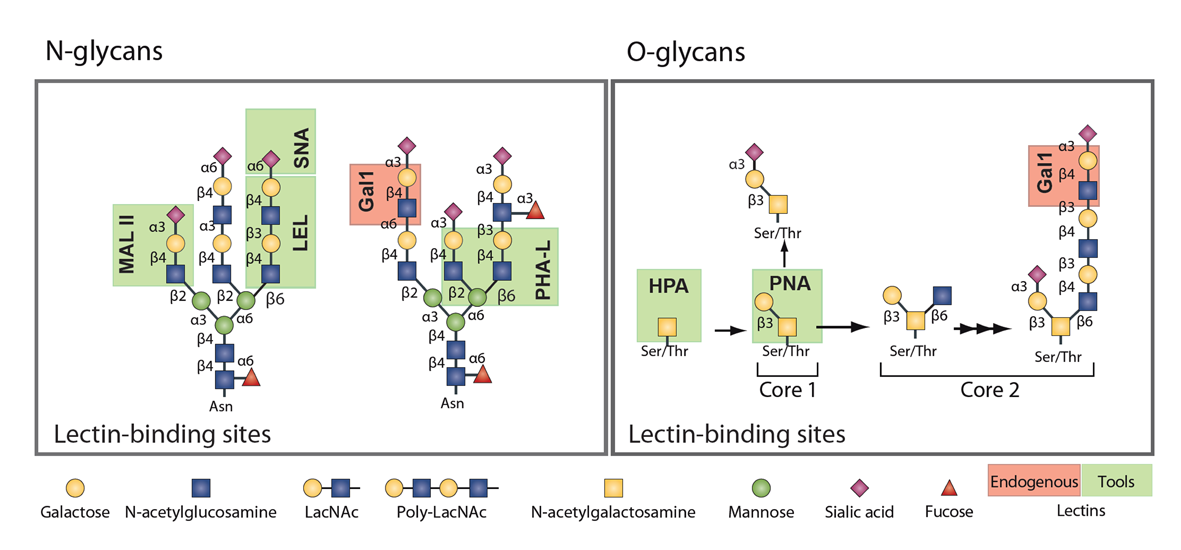

Supplement: S2 Fig — MAL II: Maackia amurensis agglutinin II; SNA: Sambucus nigra aglutinin¸ LEL: Lycopersicon esculentum agglutinin; PHA-L: Phytohemagglutinin-L; HPA: Helix pomatia agglutinin; Gal–1: galectin–1. (TIF) [file pntd.0004148.s002.tif]

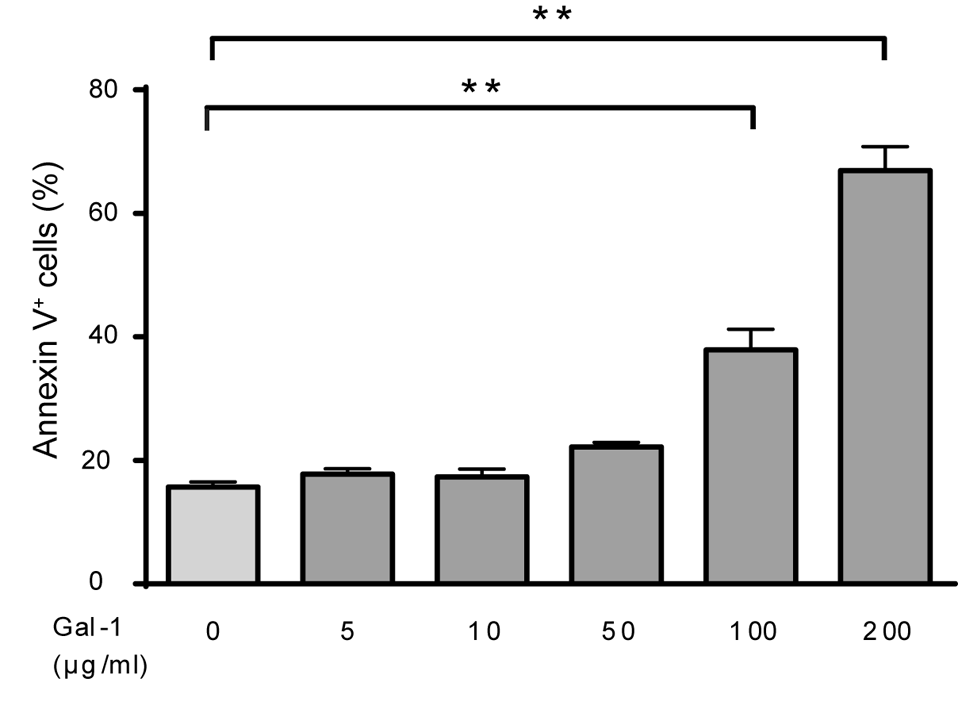

Supplement: S3 Fig — HL–1 cells were incubated with rGal–1 for 18 h, staining with FITC-Annexin-V and processed by flow cytometry. Results expressed as mean ± SEM, are representative of 2 independent experiments. Statistical analysis was performed by using ANOVA one-way followed by Tukey. **p<0.01. (TIF) [file pntd.0004148.s003.tif]

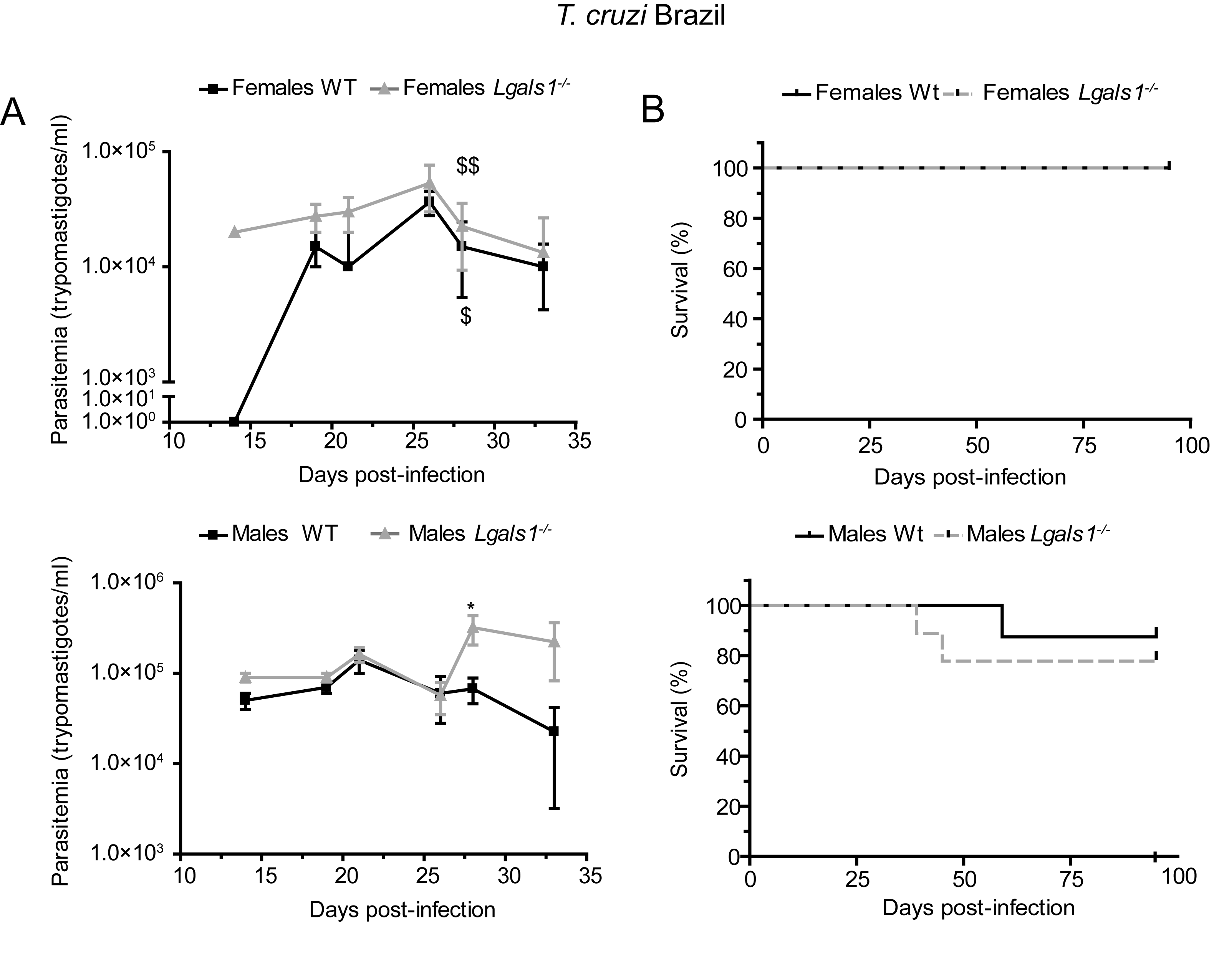

Supplement: S4 Fig — For parasitemia levels, each point represents the mean ± SEM of 5–15 animals per group, and statistical analysis was performed using Mann-Whitney U test. *p<0.05 vs. WT mice; $$ p<0.01 vs. male mice. For survival rate, statistical analysis was achieved with Log-rank test. (TIF) [file pntd.0004148.s004.tif]
