# Supplementary material for: Galectin-1 Prevents Infection and Damage Induced by Trypanosoma cruzi on Cardiac Cells
Source: PLoS Negl Trop Dis. 2015 Oct 9;9(10):e0004148. doi: 10.1371/journal.pntd.0004148 (PMC4599936; doi:10.1371/journal.pntd.0004148)
Supplement: S1 Table — 1Concentration of the lectins used. (DOCX) [file pntd.0004148.s005.docx]

**Supporting Information Table 1**

**S1 Table**: **Lectins and glycan specificity**

| Lectin | Abbreviation | Glycan specifity | Conc.^1^ (µg/ml) |
| --- | --- | --- | --- |
| *Peanut* agglutinin | PNA | Galactose β(1-3)-N-acetylgalactosamine | 10 |
| *Sambucus nigra* agglutinin | SNA | Sialic acid in α(2-6) linkage | 4 |
| *Helix pomati a*agglutinin | HPA | α-N-acetylgalactosamine present in  *O-*glycans | 20 |
| *Lycopersicon esculentum* agglutinin | LEL | Poly-lactosamine | 2 |
| Phytohemagglutinin-L | PHA-L | Branched complex *N-*glycans with lactosamines linked to mannose in a β(1-2) and β(1-6) linkage | 2 |
| *Maackia amurensis* agglutinin II | MAL II | Sialic acid in α(2-3) linkage | 20 |
| Galectin-1 | Gal-1 | Terminal N-acetyllactosamine | 0-50 |
