# Supplementary material for: Galectin-1 Prevents Infection and Damage Induced by Trypanosoma cruzi on Cardiac Cells
Source: PLoS Negl Trop Dis. 2015 Oct 9;9(10):e0004148. doi: 10.1371/journal.pntd.0004148 (PMC4599936; doi:10.1371/journal.pntd.0004148)
Supplement: S2 Table — aParasitemia is shown as median (rank) of parasites/ml of blood from 5 to 15 mice per group of each gender, at the peak. bSurvival time is shown as means ± SEM. ND: not detectable. (DOCX) [file pntd.0004148.s006.docx]

**Supporting Information Table 2**

**S2 Table**: ***T. cruzi* infection in C57BL/6 WT and *Lgals1^-/-^* mice**

|  | | |  |  |
| --- | --- | --- | --- | --- |
| *T. cruzi*  strain | Mice | Parasitemia^a^ (trypomastigotes/ml x 10^-4^) | Survival Time^b^ (days) | Mortality rate  (%) |
| Tulahuén | Females *Lgals1^-/-^* | 540 (98-610) | 22.2 ± 2.4 | 90.9 |
|  | Females WT | 30 (20-280) | 27 | 37.5 |
|  | Males *Lgals1^-/-^* | 690 (340-1052) | 20.6 ± 2.9 | 86.7 |
|  | Males WT | 290 (140-420) | 22.5 ± 4.1 | 80 |
| Brazil | Females *Lgals1^-/-^* | 3 (3-10) | ND | 0 |
|  | Females WT | 4 (2-5) | ND | 0 |
|  | Males *Lgals1^-/-^* | 16 (12-66) | 42 ± 4.2 | 22.2 |
|  | Males WT | 5 (4-13) | 59 | 12.5 |
